# Shared Leadership and Improvisation: Dual Perspective of Cognition-Affection

**DOI:** 10.3390/bs13030265

**Published:** 2023-03-17

**Authors:** Dixuan Zhang, Xiaohong Wang, Shaopeng Zhang

**Affiliations:** School of Economics and Management, Harbin Institute of Technology, Harbin 150001, China

**Keywords:** shared leadership, improvisation, cognitive flexibility, emotional intelligence, promotion focus

## Abstract

Improvisation is an effective way to cope with rapid changes and obtain unexpected opportunities in a complex environment. Based on the cognitive-affective system theory, this study investigates the dual mediating role of cognitive flexibility and emotional intelligence between shared leadership and improvisation and the moderating role of promotion focus. We used multilevel and multi-sourced data to test the theoretical model and used a social network approach to measure shared leadership in teams. Our sample was comprised of 40 teams and 240 team members. The empirical findings indicated that cognitive flexibility and emotional intelligence mediated the relationship between shared leadership and improvisation; promotion focus moderated the relationship between shared leadership and improvisation, and the mediation effect via cognitive flexibility. This study contributes to expanding on improvisation research from the perspective of shared leadership and incorporating both the cognitive and the emotional process of the generation of improvisation into a theoretical framework from a compound perspective, which will open the black box for the mediation mechanism from shared leadership to improvisation. Furthermore, promotion focus is introduced into the research and creatively corresponds to the cognition-affection mediation mechanism, which expands the applicable scope of the regulatory focus theory.

## 1. Introduction

Faced with the impact of an uncertain environment such as COVID-19, if enterprises do not take action to prevent and control in time, they may miss the opportunity or be completely submerged in the new round of reform. Research shows that improvisation is an effective way to cope with rapid changes and seize unexpected opportunities in a complex and challenging environment [1,2]. Improvisation is defined as a behavioral process in which team members try to achieve their goals in creative, immediate, and new ways, and it occurs when novel actions are deliberately designed and executed in the same process [3,4]. Improvisation brings about novel and useful solutions in the spur of the moment and helps individuals to cater to the need for rapid responses [5,6]. Meanwhile, teams offer faster and more flexible mobility and information processing capabilities than a centralized organizational structure [7,8].

Improvisation is particularly important to both scholars and practitioners in that it represents the possible response of individuals and organizations to urgent problems [9]. This behavior emphasizes an informal reaction to the status quo and an attempt to challenge organizational practices [10,11], so it is risky for followers to engage in improvisation. Therefore, the inclusiveness and support of leadership is the basic condition for followers to actively implement improvisation. However, due to its complexity, improvisation remains challenging to grasp and the important gap in this literature is still obvious [3,10]. Extant research on improvisation mainly focused on the effect of the team situation and individual traits in shaping individual and team improvisation [12,13,14], while less emphasis is placed on leadership, which plays a pivotal role in creating conditions that enable teams to be effective [1,9,15]. Moreover, the practice of informal leadership has become more widespread in current organizations as the emphasis on teammate cooperation and coordination is increasing [16,17]. Organizational scholars have argued that flexible team interaction patterns promote efficient team responses to unexpected situations [18,19]. Shared leadership, which is unlike traditional vertical leadership, may play an important role in triggering improvisation. Shared leadership distributes leadership rights and responsibilities and provides knowledge and information resources for team members to locate and share, which can satisfy their autonomy and creativity in work [16,20]. This coincides with improvisation. Therefore, we argue that shared leadership is an effective way to motivate improvisation and be responsive to the unexpected environment.

Improvisation is not just a rational process; it is also a process with emotion as the carrier. It often requires individuals to make decisions and act on intuition, which is mixed with past cognitive and emotional experiences [21]. In the 1990s, Mischel and Shoda proposed the cognitive-affective system theory, which holds that there are two processes between original information and behavior [22]. One is the encoding process, in which the original information is input into the cognitive-affective unit for encoding and interpretation. The second is the process of behavior generation, which produces different cognitive, emotional, and behavioral results through the interaction of cognitive-affective units. This theory emphasizes that individuals’ responses to situations are not passive, obedient, or indifferent. It believes that people are active and goal-oriented and that they will make plans and changes by themselves. These basic assumptions are consistent with the basic assumptions of improvisation research [9,23]. Just as Barrett et al. argued, when organizational environments are fluid, it is impossible for individuals to wait for things to be solved [24]. The significance of improvisation is not a passive resignation to bewildering complexity, but is rather an appreciative recognition that we begin not from a clean slate, but with the complexity, history, coworkers, and uneven resources at hand. In this study, individuals and their teams are not only regarded as units of rational analysis, but also as an organism combining rationality and sensibility. Thus, from a dual path of cognition and affect, this current study investigates the influence mechanism of shared leadership on improvisation.

Although scholars in the field of shared leadership readily acknowledge that there is a positive correlation between shared leadership and individual outcomes [17,25,26,27], studies show that there is a large unexplained variance in the impact of shared leadership on individual outcomes. Previous research has found that cognitive and affective processes are moderated by promotion focus [28]. The influence of shared leadership on individual improvisation may be related to individual characteristics, such as promotion focus. Promotion focus is a self-regulation inclination, which is connected to self-enhancement demands. Gorman et al. found that the proper matching of leadership and individual regulatory focus can improve the effectiveness of leadership [29]. However, previous research has pointed out that promotion focus is positively related to improvisation [2,21]. Members with high promotion focus are more sensitive to the occurrence of positive results and are more active in their work. Based on regulatory focus theory, promotion focus will urge team members to change their reactions and behaviors in order to achieve their goals [30]. In an emergency, whether an individual can immediately produce improvisation is closely connected with promotion focus. Thus, whether promotion focus has a significant impact in influencing the relationship between shared leadership and improvisation, and in determining the relationship between shared leadership and cognition-affection paths is the third research question in the current study.

Accordingly, we constructed and tested a theoretical model that examined how shared leadership may influence improvisation via cognitive-affective dual paths and aimed to make three significant contributions. First, this paper expands upon improvisation research from the perspective of leadership and explores the cross-level impact of shared leadership on improvisation. Mannucci et al. emphasized that improvisation is not a given but needs to be developed [13]. Leadership, as a pivotal role in creating conditions that enable teams to be effective, has not attracted enough attention to its impact on individual improvisation [31,32]. Second, based on cognitive-affective system theory, this paper supplements the mediation mechanism from shared leadership to improvisation. Third, this paper identifies promotion focus as an important boundary condition that moderates the impact of shared leadership on improvisation and the impact of shared leadership on improvisation via cognitive flexibility, thus underscoring the importance of promotion focus for improvisation for it may compensate for the lower degree of cognitive flexibility.

## 2. Theoretical Background and Research Hypothesis

### 2.1. Cognitive-Affective System Theory

In the 1990s, Mischel and Shoda proposed the cognitive-affective system theory, which holds that there are two processes between original information and behavior [22]. One is the encoding process, in which the original information is input into the cognitive-affective unit for encoding and interpretation. The second is the process of behavior generation, which produces different cognitive, emotional, and behavioral results through the interaction of cognitive-affective units. This theory emphasizes that individuals’ responses to situations are not passive, obedient, or indifferent [14]. It believes that people are active and goal-oriented and that they will make plans and changes by themselves. These basic assumptions are consistent with the basic assumptions of improvisation research [9,23]. Therefore, in this paper, individuals and their teams are not only regarded as units of rational analysis, but also as an organism combining rationality and sensibility, and the formation mechanism of improvisation is observed through the establishment of cognition-emotion two paths.

### 2.2. Shared Leadership and Improvisation 

Individual improvisation highlights that team members spontaneously integrate or utilize existing resources and use creative methods to effectively solve emergent problems when unexpected environmental changes cannot be dealt with by pre-planning [10]. The importance of improvisation is not a passive resignation to bewildering complexity, but is rather an appreciative recognition that individuals begin not from a clean slate, but with the complexity, history, coworkers, and uneven resources at hand when organizational environments are fluid [19]. However, improvisation emphasizes that individuals need to go beyond just following procedures and executing strategic plans to quickly adapt to new circumstances [11], so it is risky for followers to engage in improvisation. Therefore, the inclusiveness and support of leadership is the basic condition for followers to actively implement improvisation. In current organizations, informal leadership has grown more prevalent due to the increased focus on teammate cooperation and coordination [16,17]. Shared leadership, which is unlike traditional vertical leadership, may play an important role in triggering improvisation.

Different from the traditional centralized structure in which only one person performs the leader role, shared leadership enables team members to switch between the roles of “leader” and “follower.” Shared leadership distributes leadership rights and provides information for team members, which satisfies their autonomy and creativity in work and enhances their competence motivation to implement improvisation [14,32]. On the one hand, shared leadership meets team members’ need for supporting resources in their work and effectively supplements the resources consumed by adopting new methods to solve problems [33,34], which helps improve their perception of the ability to solve emergent problems [14]. Individual positive recognition of their own ability not only helps to reduce their sensitivity to uncertainty and strengthens the confidence to overcome difficulty, but can also make them focus on things worth pursuing, so as to not be bound by various rules within the organization [35]. This process enhances team members’ motivations to go out of their comfort zone and explore the unknown, thus approaching improvisation by creatively dealing with emergencies. On the other hand, shared leadership encourages team members to actively exert their abilities, supports members to involve in team decision-making, and advocates for members to express their new ideas [23]. Therefore, when faced with unexpected situations, team members will be willing to give full play to their creative talents and take the initiative to solve problems by improvisation.

Although competence motivation is very important, even if individuals feel capable of carrying out improvisation, it is not necessary to carry out the behavior. Individuals also need a strong cause motivation to carry out improvisation. Shared leadership encourages team members to take the initiative to assume the “leader” role, enhances their perception of self-efficacy, and meets their autonomy needs [36,37]. Therefore, team members will show a positive attitude towards participation in their work and take the initiative to implement improvisation to help the team get rid of difficulties. Therefore, we hypothesize:

**H1.**  *Shared leadership is positively related to team members’ improvisation*.

### 2.3. Cognitive Path: The Mediating Role of Cognitive Flexibility

Based on the cognitive-affective system theory, the formation mechanism from shared leadership to improvisation is observed through the establishment of cognition and emotion two paths. In the transition from shared leadership to improvisation, the first step is to rely on cognition to understand and interpret the environment. Cognitive flexibility (CF) is an individual’s ability to change and switch cognitive sets in order to adapt to the stimuli of environmental changes [38]; it can break individual behavioral inertia and solve problems spontaneously and creatively.

Specifically, first, shared leadership provides space for team members to think independently and empowers them to fully deconstruct their work cognitive patterns [37]. CF helps members to display diversified cognitive resource-allocation abilities in this process, so as to match with the leadership structure of the team and stimulate team members’ high innovation beliefs and improvisation behavior [39,40]. In contrast, team members with low CF were more focused on the difficulty of the task itself and how to avoid failure by minimizing risk, thus making it difficult to engage in innovative activities [38].

Second, CF enables team members to have strong environmental perceptions and be willing to believe that they can flexibly adapt to new situations [39,40]. Under the shared leadership structure, team members are required to independently handle tasks and responsibilities. In this process, there will be many unexpected situations planned in advance. At this time, CF will make team members’ thinking more flexible and insightful [38], as well as make them able to quickly respond to innovation tasks, have high innovation belief, and thus show improvisation behavior.

Finally, CF can help team members quickly adapt to the “leader-follower” role transition in the shared leadership process, and thus quickly shift perspectives to solve new problems and adapt to changes in the environment. Shared leadership requires team members to quickly change roles between “leader” and “follower” according to team tasks [31,41]. Team members can quickly adapt to this process by virtue of CF and try various new schemes and paths, which is conducive to stimulating improvisation [38].

Consequently, we propose the following hypothesis:

**H2a.**  *Cognitive flexibility mediates the relationship between shared leadership and improvisation*.

### 2.4. Affective Path: The Mediating Role of Emotional Intelligence

Individuals differ in their ability to withstand anxiety or insecurity in response to environmental changes or challenges [42]. Emotional intelligence (EI) refers to the ability of an individual to control his or her own emotions and those of other members, distinguish the differences between different emotions, and guide his or her thinking and actions through different emotional information [42,43]. EI plays a valuable role in effectively processing information in changing environments [43,44]. Thus, we indicate that shared leadership may stimulate improvisation through EI based on cognitive-affective system theory.

Different from traditional vertical leadership, shared leadership encourages team members to spontaneously and actively participate in the management of the team, assume leadership responsibilities, and seek solutions to problems, which can motivate team members to shape their matching emotional cognition, understanding, application, and management abilities, so as to improve their EI [45]. EI enables team members to flexibly use emotional resources in dynamic and complex innovative activities and propose new ideas according to different situations, which is conducive to the continuous generation of improvisation. In this process, EI will strengthen the exchange of team members’ ideas and information with other members in a shared and interactive attitude and can also effectively promote them to take the initiative to implement improvisation [46].

Shared leadership provides a space for team members to think independently and empowers them. During this process, EI helps team members efficiently deal with conflicts and tensions in order to break through the existing rules and constraints of the organization, thus allowing them to deviate from established conventions to engage in improvisation [47]. Shared leadership provides free space for team members to think independently and empowers them to fully deconstruct their working mode so that team members tend to conduct emotional management actively rather than passively, thus helping to shape EI. Meanwhile, EI encourages team members to be more creative and keeps team members engaged in a continuous positive state [36,45]. Positive traits make such individuals more focused on problem-solving rather than reasoning about problems, which can make sure that team members are constantly thinking and using more flexible solutions when faced with unexpected problems [26,46].

Thus, we hypothesize:

**H2b.**  *Emotional intelligence mediates the relationship between shared leadership and improvisation*.

### 2.5. The Moderating Role of Promotion Focus

Promotion focus (PF) comes from the regulatory focus theory proposed by Higgins in 1997 [48]. This theory suggests that people regulate their behavior through two different systems: promotion focus and prevention focus. PF is the individual trait that develops during growth, which influences behaviors [49,50]. It leads individuals to be concerned with attaining positive outcomes without considering the possible negative outcomes [48]. Recent research has shown that regulatory focus theory is regarded as an appropriate framework for understanding individual improvisation and members with high promotion focus are willing to risk and take active ways to accomplish goals [51,52]. Contrarily, members with low promotion focus exhibit a preference for low risks and are sensitive to the negative aspects of tasks [53]. Improvisation implies that it will break the normal procedure and has some risks [14]. Therefore, when facing emergencies, members with high promotion focus are actively motivated to adapt to the circumstances. That is, members will judge and weigh the aftermath of improvisation, and higher promotion focus will urge members to improvise as a complement to the individual orientation [53]. Thus, the level of promotion focus may influence the impact of shared leadership on improvisation. Members with high promotion focus are highly concerned about positive outcomes and actively seek out latent opportunities, so they dare to challenge the rules and are more likely to implement improvisation [26,50].

Based on regulatory focus theory, individuals can achieve set goals by controlling their reactions and thoughts [50]. Before implementing improvisation, shared leadership will largely activate team members’ cognitive and affective units (cognitive flexibility and emotional intelligence). When team members have a high promotion focus, shared leadership can transfer the indirect effect on employee improvisation through cognitive flexibility and emotional intelligence. Thus, it is logical in the current paper to propose that promotion focus moderates the indirect effects of shared leadership on improvisation.

From the perspective of cognitive path, under the shared leadership structure, members with high promotion focus are inclined to adopt a holistic and divergent cognitive processing method and widely collect information, which is conducive to identifying opportunities in the environment and easy-to-ignore risk information [34,53]. Therefore, they are more inclined to implement improvisation. In addition, members with high promotion focus prefer to adopt flexible, exploratory, and innovative cognitive styles when facing external stimuli. Therefore, the match of promotion focus and cognitive flexibility will produce effective adjustment, thus promoting the generation of improvisation [38,50]. Conversely, members with low promotion focus tend to generate cognition, experience doing more, make more mistakes, and lose achievement motivation, so they will not produce creative improvisational behavior [3,53].

From the perspective of affective path, promotion focus enhances team members’ sense of participation and emotional intelligence by cultivating their autonomous learning ability, thus stimulating more creative behaviors under the shared leadership structure [37,47]. Members with high promotion focus are fully energetic and passionate when pursuing their goals [42,45]. This positive emotion makes them focus on the win-win situation of individuals and teams when addressing challenges, so as to actively implement improvisation [41]. Contrarily, members with low promotion focus are more prone to fear and anxiety and are unable to achieve a relaxed and calm state of mind, thus affecting the play of creative improvisation [5,12].

Therefore, the following hypothesis is put forward:

**H3a.**  *Promotion focus moderates the relationship between shared leadership and improvisation, such that the relationship is stronger when there are higher rather than lower levels of promotion focus*.

**H3b.**  *Promotion focus moderates the mediated relationship between shared leadership and improvisation* via *cognitive flexibility, such that the relationship is stronger when there are higher rather than lower levels of promotion focus*.

**H3c.**  *Promotion focus moderates the mediated relationship between shared leadership and improvisation* via *emotional intelligence, such that the relationship is stronger when there are higher rather than lower levels of promotion focus*.

**Figure 1:**
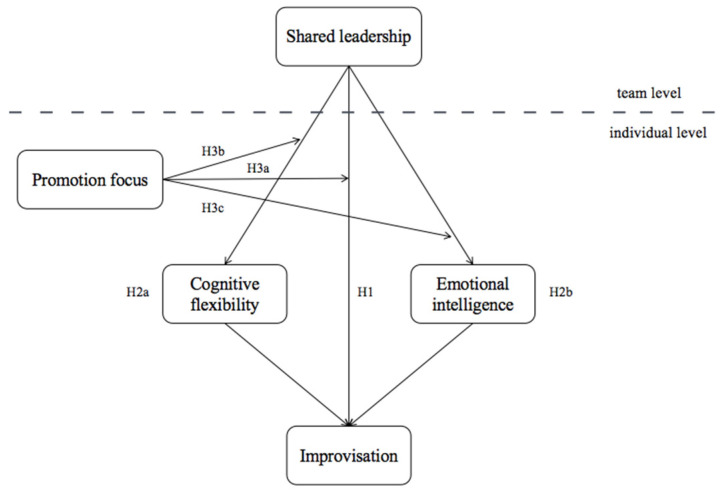
Theoretical model.

Figure 1 depicts our theoretical research model.

## 3. Methods

### 3.1. Participants and Procedure

The sample for our study included 40 teams and 240 team members from China. Firstly, through alumni resources, we identified 70 companies that complete tasks in the form of teams, which involved the Internet, finance, electronics, manufacturing, and service industries. Each company provided a team composed of 4–10 members and these members have to coordinate the team’s task plan with each other, so as to form a self-management work team. Before the formal survey, we conducted a semi-structured online interview with one representative from each team to further identify leadership patterns among team members. After confirming the samples’ shared leadership structures, we decided to collect a three-wave dataset. At the first stage (T1), we asked team members to record their responses on demographic variables, shared leadership, and team knowledge sharing. In the first stage of the survey, we sent 70 teams the questionnaires and effectively collected 56 teams. We numbered each valid questionnaire to facilitate the second wave. One month later, at the second stage (T2), we re-contacted the previous respondents and invited them to report their responses on cognitive flexibility and emotional intelligence. In the second stage, we collected 47 teams’ valid questionnaires from the same teams. At the third stage (T3), with a one month gap, we continued to send questionnaires to the teams from the first and second stages of feedback, and finally, 43 teams submitted their responses on improvisation. After excluding invalid questionnaires, the final effective data consisted of 40 teams and 240 individual members. With the help of alumni, we ensured a 90% response rate for each team, thus meeting the needs of calculating network analysis [54]. The average team size of the sample was 6. Of the participants, 51.67% were male, 48.33% were female, 22.17% held a bachelor’s degree, 39.58% held a master’s degree, and 21.25% held a Ph.D. degree.

### 3.2. Measures

#### 3.2.1. Shared Leadership

Shared leadership was measured by a social network approach within each team, which was developed by Mathieu et al. [55]. They contend that a higher density corresponds to a greater level of shared leadership. We asked each member to rate each of his/her teammates on a single-item scale: “To what degree does your team rely on this individual for leadership?” on a 7-point Likert scale, summed all of the values, and divided them by the total possible number of ties among team members. Equation (1) shows how to calculate the density for shared leadership:

*Density* = *S*/*N*(*N* − 1) (1)

In this equation, *S* is the sum of all values that team members would rate each other for leadership. *N* equals the number of team members; *N*(*N* − 1) is the total number of possible ties in a team.

We followed the previous measurement approach in social network literature to measure shared leadership [25,27,55]. Since shared leadership represents the distribution of leadership among members in a team, the measure of density with all members rating each other’s leadership appropriately captures the nature of shared leadership [36,41,55].

#### 3.2.2. Cognitive Flexibility

We used 8 items developed by Martin and Rubin to measure cognitive flexibility, including “I can communicate the same idea in many different ways”, “I can find workable solutions to seemingly unsolvable problems”, etc. [56]. The Cronbach’s alpha coefficient for this scale was 0.776 (See Table 1). CF assesses an individual’s awareness that different viewpoints and behaviors exist in a given context, besides an individual’s readiness and self-efficacy to consider these different choices [39]. 

#### 3.2.3. Emotional Intelligence

Drawing on the emotional intelligence scale developed by Goleman, we included a total of 8 items, such as “I have a good understanding of my own emotions” and “I always know others’ emotions from their behavior” [57]. Each item of the Goleman Emotional Intelligence Questionnaire indices a situation of life that the subject must put him/herself in that position and select one of the options that are more compatible with his/her mental and psychological situation [40,41]. The Cronbach’s alpha of the scale in this study was 0.845.

#### 3.2.4. Improvisation

We used a 7-item scale by Vera and Crossan and the sample items were “I deal with unanticipated events on the spot” and “I respond at the moment to unexpected problems” [58]. The robustness of our method to assess improvisation is twofold. First, the measurement of improvisation through a Likert-scale directly administered to the subjects of the study is a consolidated way of considering improvisation in quantitative research [9,16,17]. Second, the scale has been proven to be robust by several studies and across different settings [9,58]. The Cronbach’s alpha of the scale in this study was 0.906.

#### 3.2.5. Promotion Focus

The PF measurement scale was from Wallace and Chen. This scale was composed of 6 items and the sample items are “My focus is how do I succeed” and “I often think about what kind of person I will be in the future” [59]. The scale we adopted for assessing PF has been proven to be robust by previous studies and across different settings [60,61,62]. The Cronbach’s alpha of the scale in this study was 0.902.

#### 3.2.6. Control Variables

As suggested by previous research, we used team members’ gender, age, and educational background as controls in individual level, teams’ number of members, and time of establishment as controls in team level.

## 4. Data Analysis and Results

### 4.1. Data Aggregation

Shared leadership is a team-level variable, but this variable was rated by individual members, therefore, we justified aggregation to the team level by calculating the values of rwg, ICC(1), and ICC(2). The results provided justification for aggregation for shared leadership (rwg = 0.84, ICC(1) = 0.294, and ICC(2) = 0.729), which supported the aggregation of individual team member responses to generate a team-level measure.

### 4.2. Confirmatory Factor Analysis

Anderson and Gerbing proposed a two-step SEM analysis with maximum likelihood estimation and bootstrapping to assess the measurement model [63]. The first step focused on measurement validity through confirmatory factor analysis (CFA) and the second step focused on assessing structural relationships simultaneously for hypothesis testing. We performed a number of confirmatory factor analyses to examine the distinctiveness of key variables. The overall model fit was assessed by χ^2^/df, CFI, TLI, RMR, and RMSEA. Table 2 shows that the hypothesized five-factor model (including SL, CF, EI, PF, and IM) indicated a good fit to the data (χ^2^/df = 2.485, CFI = 0.865, TLI = 0.842, SRMR = 0.091, RMSEA = 0.079). Based on Hu and Bentler’s two-index presentation strategy and relevant research [64], our value of SRMR indicated an acceptable fit to the data, our value of χ^2^/df indicated a good fit to the data, and values of RMSEA, CFI, and TLI indicated an acceptable fit to the data [17,65,66,67,68]. We also compared the five-factor model with the four-factor model, in which CF and EI were loaded on one factor; with the three-factor model, in which CF, EI, and PF load on one factor; with the one-factor model, in which all items load on one factor. However, these three models did not fit the data well. In order to investigate and confirm the convergent validity and discriminant validity of each variable, we calculated the average variance extracted (AVE) values and composite reliability (CR) values of four variables to examine convergent validity. Table 1 shows that the CR is greater than 0.7 and the AVE is greater than 0.5, suggesting a decent convergent validity [64,69]. The square roots of the AVE values of the four variables were all greater than their correlation coefficients, indicating that the discrimination validity is good.

### 4.3. Common Method Bias

Given that all scales we administered were self-reported, there was a risk of common method bias [70]. Therefore, we checked for possible common method bias using Harman’s single-factor test. The test results indicated that the first factor in the exploratory factor analysis only explained 35.036% (<40%) loading, proving the absence of common method bias [71]. Thus, the results indicated that the common method bias was unlikely to threaten our study.

### 4.4. Descriptive Statistics

The means, standard deviations, correlations, and reliability of the variables at the individual and team levels of analysis are presented in Table 3, respectively. As we expected, SL is positively associated with CF, EI, and IM; CF and EI are positively associated with IM; PF is positively associated with IM. Our hypothesized relationships are initially supported by correlations among variables.

### 4.5. Hypotheses Testing

We used Mplus version 7.0 to test the cross-level hypotheses. As displayed in Table 4, after including all the control variables, SL has a significant impact on IM (Model 1: β = 0.593, *p* < 0.001), which supported H1. We applied bootstrapping to test the mediating effect of CF and EI, and the results of 5000 Monte Carlo replications showed that the indirect effects of SL on the dependent of IM through CF and EI were significant. The 95% confidence intervals were [0.061, 0.313] and [0.021, 0.250], excluding zero (see Table 5). Hence, H2a and H2b were supported.

**Figure 2:**
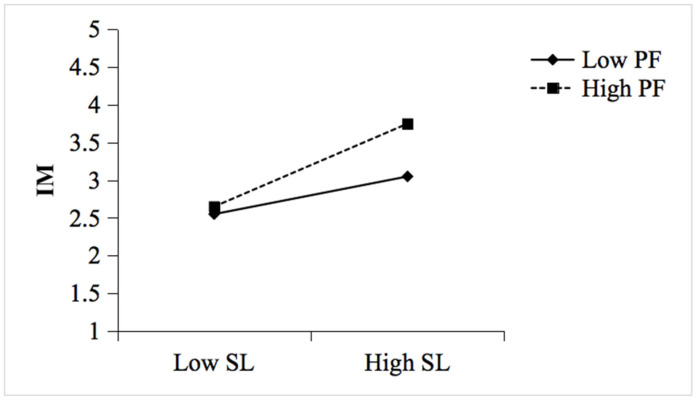
The moderating role of PF on the relationship between SL and IM.

H3a, H3b, and H3c tested the cross-level moderating effects of PF. As shown in Table 4, after including all the control variables, the interaction term of SL and PF on IM was significant (Model 4: β = 0.306, *p* < 0.001), which supported H3a. H3b predicted that PF would moderate the indirect impact of SL on IM via CF. The results revealed that the indirect effect of SL on IM was stronger at low levels of PF than at high levels (see Table 6), which was contrary to expectations in that PF mattered more when CF was lower than when it was higher. Hence, H3b was not supported. H3c proposed that PF would moderate the indirect impact of SL on IM via EI. Then, we calculated the magnitude of the indirect effect on different levels of PF. As shown in Table 6, the indirect influence of SL on IM through EI was not significant at high PF (SE = 0.014, 95% CI = [−0.089, 0.250]) containing 0 nor low PF (SE = 0.011, 95% CI = [−0.021, 0.103]) containing 0. Hence, H3c was not supported. In order to more clearly reveal the moderating effects of PF, we conducted a simple effect analysis based on the suggestions of Liu et al. [72], as shown in Figure 2 below.

## 5. Discussion

Based on cognitive-affective system theory, we examined how shared leadership motivates improvisation by highlighting the mechanisms through which such influence occurs. First, in line with our hypothesis, shared leadership has a significantly positive impact on improvisation. The positive impact can be analyzed from two perspectives. On the one hand, from the perspective of competence motivation, shared leadership distributes responsibilities and provides information resources for team members to locate and share, which can satisfy their autonomy and creativity in work and enhance their competence motivation to implement improvisation [6]. On the other hand, from the perspective of cause motivation, shared leadership encourages team members to take the initiative to assume the “leader” role, enhances their perception of self-efficacy, and meets their autonomy needs, which can encourage members to implement improvisation to help the team get rid of difficulties [8,13].

Second, based on cognitive-affective system theory, we found that CF and EI play dual mediating roles between shared leadership and improvisation. In the transition from shared leadership to improvisation, the first step is to rely on cognition to understand and interpret the environment. CF helps members to display diversified cognitive resource-allocation abilities in this process, so as to match with the leadership structure and stimulate team members’ high innovation beliefs and improvisation behavior [31,33]. Meanwhile, different individuals differ in their ability to withstand anxiety or insecurity in response to environmental changes or challenges. EI plays a valuable role in effectively processing information in changing environments [41,73]. Thus, we indicated and examined that shared leadership may stimulate group improvisation through EI.

Third, we found that promotion focus moderates the relationship between shared leadership and improvisation; promotion focus moderates the mediated relationship between shared leadership and improvisation via CF, but the cross-level effect was contrary to expectations in that PF mattered more when CF was lower than when it was higher. This may be because PF may compensate for the lower degree of cognitive flexibility [48]. Based on regulatory focus theory, PF causes individuals to pay more attention to their ideals and hopes, as well as be more flexible [52]. When confronted with external cues, members with strong promotion focus are likely to adopt flexible, exploratory, and innovative cognitive styles [51]. Therefore, the moderated mediation association between SL and IM via CF is stronger under a lower promotion focus and weaker under a higher promotion focus. However, we did not find evidence for the moderated mediating effect of PF on the shared leadership–improvisation relationship via EI. Compared with CF, EI is more stable and relatively independent [74,75]. Therefore, compared with the mediating effect of CF, the mediating effect of EI is not easily regulated by PF. These findings still need further investigation.

### 5.1. Theoretical Contributions

First, this paper expands upon improvisation research from the perspective of leadership and explores the cross-level impact of shared leadership on improvisation. 

Improvisation focuses on an informal reaction to the status quo and an attempt to challenge organizational practices [3,11], so it is risky for followers to engage in improvisation. Therefore, the inclusiveness and support of leadership is the basic condition for followers to actively implement improvisation. However, not much is known about the role of leadership in the process of improvisation, as well as about the type of leadership that will enhance or motivate improvisation. Although leadership is a much-studied phenomenon, it has been of little concern to those researching improvisation, perhaps in part because the latter phenomenon is still in its initial stage of development. Furthermore, studies that concentrate on the role of leadership on individual improvisation are still in their infancy [3,75]. This absence is somewhat surprising because improvisation is the creative behavior of individuals to solve unplanned problems in time, which requires a rapid transition from plan to implementation [58]. Whether at the enterprise or group level, there may be delays or weakening of improvisation behavior due to too much emphasis on cooperation, which reduces the infinite possibility of independent individuals. Meanwhile, previous research on improvisation usually focuses on factors at a single level, while Mangi et al. believe that research on improvisation should adopt a cross-level analysis method to consider the influence mechanism of team factors on individual improvisation [23]. In view of this, the current study explores the cross-level impact of shared leadership on improvisation, which responds to the scholars in the call for study across levels of improvisation and deepens the understanding of individual behavior triggers off the cuff [6,10,11].

Second, based on cognitive-affective system theory, this study supplements the mediation mechanism from shared leadership to improvisation. Most existing studies discuss the formation mechanism of improvisation from a single perspective [76,77,78]. This study incorporates both the cognitive and the emotional process of the generation of improvisation into a theoretical framework from a compound perspective, which will open the black box for the mediation mechanism from shared leadership to improvisation.

Third, we examine PF as a crucial boundary condition that moderates the relationship between shared leadership and improvisation as well as moderates the relationship between shared leadership and CF for improvisation. Regulatory focus theory has been widely used in social psychology research, but it has not been a concern to organizational management scholars until recent years. In this paper, promotion focus is introduced into the research of shared leadership and individual improvisation and creatively corresponds to the cognition-affection mediation mechanism, which expands the scope of application of regulatory focus theory. Meanwhile, research shows that there is a large unexplained variance in the effect of shared leadership on individual outcomes, however, Gorman et al. and Lanaj et al. indicate that regulatory focus theory has unique explanatory power on individual behavior through meta-analysis [29,78]. Consistent with their findings, we identify that PF is potent, such that a high level of PF amplifies the positive effect according to our moderated mediation model.

### 5.2. Practical Contributions

First, enterprises should strengthen the leadership training of employees, build a team structure and shared leadership within the team, and develop leadership training programs through a variety of ways and means. The realization of improvisation behavior is closely related to the solution of complex and uncertain problems. In this context, shared leadership, which emphasizes the sharing of leadership roles and responsibilities among team members, fits this need well. This is because shared leadership not only emphasizes that members take the initiative to solve complex problems, but also emphasizes that members should be given the authority needed to solve these problems, which means “let those who can hear the fire make decisions”.

Second, the key point of shared leadership in promoting team members’ improvisation is to grasp team members’ cognitive and emotional patterns. The motivational effect of shared leadership requires a comprehensive understanding of the psychological transmission patterns of team members in the face of shared leadership structures. On the one hand, the compound cognitive pattern of team members should be shaped to improve the effect of shared leadership. On the other hand, cultivating a high level of emotional intelligence among team members helps them deal with risks and uncertainties in the innovation process with positive and stable emotions.

Third, the effect of shared leadership on improvisation varies with the individual’s promotion focus trait. Team members with strong promotion focus should be delegated to more appropriately and given more flexibility and autonomy to enhance their sense of self-efficacy and intrinsic motivation, so as to stimulate their improvisation. Although previous studies have regarded regulatory focus as a relatively stable feature, recent research has revealed that context can alter the regulatory focus [79]. Therefore, in addition to considering this factor in the arrangement of team personnel, members can also be guided to form promotion focus through training.

### 5.3. Limitations and Future Research

First, this study discusses the cross-level impact and action path of shared leadership on improvisation from the compound perspective. Although we tested the mediating role of team members’ CF and EI in the main effect from the “cognitive-emotional” compound perspective, this paper is only a preliminary attempt to study from the compound perspective, and the research can be deeply excavated based on different mediating combinations in the future. 

Second, this study only considered the regulatory effect of promoting focus, but individual behavior may also be affected by other environmental factors. Therefore, other personality traits (e.g., proactive personality, work passion, innovative efficacy), other environmental factors (e.g., team climate, team support, organizational culture), and their interactions can be considered as moderated variables in future research.

Third, this study did not include the influence of traditional Chinese culture. Traditional Chinese culture emphasizes “Zhong Yong” (The Golden Mean) and team members are more traditional, which makes them more compliant with organizational rules and unwilling to take risks in improvisation. Therefore, future research can further explore the moderating effects of team members’ values of moderation, power distance, and traditionality.

## 6. Conclusions

Based on cognitive-affective system theory, we explored the effects and mechanisms of shared leadership on improvisation. Although previous research has proposed the effect of the team situation and individual traits in shaping individual and team improvisation, less emphasis is placed on informal leadership. The results show a significant positive effect of shared leadership on improvisation, in which cognitive flexibility and emotional intelligence play mediating roles. Further, promotion focus moderated the relationship between shared leadership and improvisation, and the mediation effect via cognitive flexibility. Our findings expand upon improvisation research from the perspective of shared leadership and incorporate both the cognitive and the emotional process of the generation of improvisation into the theoretical framework. We believe that the current results will inspire future research that will excavate the relationship between shared leadership and improvisation.

## Figures and Tables

**Table 1:** Reliability and Validity Analysis.

	Items	Cronbach’s Alpha Value	CR	AVE
Shared leadership	1 item	-	-	-
Cognitive flexibility	8 items	0.776	0.892	0.509
Emotional intelligence	8 items	0.845	0.945	0.682
Improvisation	7 items	0.906	0.961	0.778
Promotion focus	6 items	0.902	0.928	0.684

**Table 2:** The result of confirmatory factor analysis.

Model	χ^2^ (df)	RMSEA	CFI	TLI	SRMR
Model A	628.82 (253)	0.079	0.865	0.842	0.091
Model B	717.74 (257)	0.102	0.685	0.639	0.186
Model C	872.44 (261)	0.119	0.776	0.737	0.167
Model D	1118.82 (267)	0.219	0.460	0.397	0.146

Model A: full measurement model; Model B: PIS and EI load on one factor; Model C: PIS, EI, and PF load on one factor, whereas other constructs represent separate factors; Model D: all items load on one factor.

**Table 3:** Means, standard deviations, and correlations among variables.

	Gender	Age	Education	CF	EI	PF	IM	Team Size	Team Age	SL
Individual level										
Gender										
Age	−0.128 *									
Education	0.037	0.041								
CF	0.002	0.005	0.002	**0.713**						
EI	−0.042	0.033	−0.062	0.291 **	**0.826**					
PF	−0.001	−0.051	0.027	0.003	−0.185 *	**0.827**				
IM	−0.053	−0.092	0.044	0.385 **	0.509 **	0.003	**0.882**			
Team level										
Team size	−0.073	0.018	−0.004	−0.085	−0.041	−0.041	0.075			
Team age	0.011	0.021	−0.061	0.023	−0.019	−0.037	0.010	−0.124		
SL	−0.027	0.031	−0.013	0.340 ^**^	0.371 **	0.033	0.595 **	0.118	0.012	
M	1.483	2.779	2.925	4.496	4.295	5.217	4.949	6.000	2.875	5.008
SD	0.501	0.741	0.830	0.923	0.706	0.996	0.872	1.179	1.186	0.993

The square root of AVE of each variable is shown in bold along the diagonal; SL represents shared leadership, CF denotes cognitive flexibility, EI represents emotional intelligence, IM is improvisation, and PF reflects promotion focus; * represents *p* < 0.05, and ** denotes *p* < 0.01.

**Table 4:** Results of regression analyses.

Variables	IM			
	Model 1	Model 2	Model 3	Model 4
Intercept	4.173 ***	4.470 ***	5.017 ***	4.935 ***
Individual level				
Gender	−0.068	−0.092	−0.105	−0.109
Age	−0.101	−0.155	−0.165	−0.165
Education	0.037	0.233	0.122	0.141
CF		0.447 ***		
EI			0.544 ***	
Team level				
Team size	−0.050	0.016	−0.032	0.111
Team age	0.022	−0.027	0.018	0.010
SL	0.593 ***	0.462 ***	0.556 ***	0.378 ***
SL × PF				0.306 ***

Note: SL represents shared leadership, CF denotes cognitive flexibility, EI represents emotional intelligence, IM is improvisation, and PF reflects promotion focus; *** represents *p* < 0.001. The values in parentheses are standard errors.

**Table 5:** Results of mediation of CF and EI in the relationship between SL and IM.

Effects	Estimate	SE	*p* Value	Boot 95% CI
SL—CF—IM	0.175	0.065	0.008	0.061, 0.313
SL—EI—IM	0.120	0.059	0.043	0.021, 0.250

Note: SL represents shared leadership, CF denotes cognitive flexibility, EI represents emotional intelligence, IM is improvisation, and PF reflects promotion focus. The values in parentheses are standard errors.

**Table 6:** Bootstrapping estimates for moderated mediation.

PF	Indirect Effect	Moderated Mediation	
SL—CF—IM		SE	Boot 95% CI
Low (−1 SD)	0.102	0.032	0.047, 0.172
Medium (mean)	0.083	0.023	0.042, 0.134
High (+1 SD)	0.064	0.013	0.024, 0.116
SL—EI—IM			
Low (−1 SD)	0.039	0.011	−0.021, 0.103
Medium (mean)	0.099	0.027	−0.053, 0.159
High (+1 SD)	0.161	0.014	−0.089, 0.250

Note: SL represents shared leadership, CF denotes cognitive flexibility, EI represents emotional intelligence, IM is improvisation, and PF reflects promotion focus. The values in parentheses are standard errors.

## Data Availability

The data that support the findings of this study are available from the corresponding author upon reasonable request.
